# Histological and Histomorphometric Analyses of Bone Regeneration in Osteoporotic Rats Using a Xenograft Material

**DOI:** 10.3390/ma14010222

**Published:** 2021-01-05

**Authors:** Marwa Y. Shaheen, Amani M. Basudan, Abdurahman A. Niazy, Jeroen J. J. P. van den Beucken, John A. Jansen, Hamdan S. Alghamdi

**Affiliations:** 1 Department of Periodontics and Community Dentistry, College of Dentistry, King Saud University, P.O. Box 2455, Riyadh 11451, Saudi Arabia; mashaheen@ksu.edu.sa (M.Y.S.); abasudan@ksu.edu.sa (A.M.B.); 2 Department of Oral Medicine and Diagnostic Sciences, College of Dentistry, King Saud University, P.O. Box 2455, Riyadh 11451, Saudi Arabia; aaniazy@ksu.edu.sa; 3 Department of Dentistry-Biomaterials, Radboudumc, P.O. Box 9101, 6500HB Nijmegen, The Netherlands; Jeroen.vandenBeucken@radboudumc.nl (J.J.J.P.v.d.B.); John.Jansen@radboudumc.nl (J.A.J.)

**Keywords:** osteoporotic condition, animal model, xenograft, bone regeneration

## Abstract

We evaluated the effect of osteoporotic induction after eight weeks of initial healing of bone defects grafted with a xenograft material in a rat model. Bone defects were created in the femoral condyles of 16 female Wistar rats (one defect per rat). The defects were filled with bovine bone (Inter-Oss) granules. After eight weeks of bone healing, rats were randomly ovariectomized (OVX) or sham-operated (SHAM). At 14 weeks of bone healing, all animals were euthanized. Bone specimens were harvested and processed for histological and histomorphometric analyses to assess new bone formation (N-BF%), remaining bone graft (RBG%) and trabecular bone space (Tb.Sp%) within the defect area. After 14 weeks of bone healing, histological evaluation revealed a significant alteration in trabecular bone in OVX rats compared to SHAM rats. There was lower N-BF% in OVX rats (22.5% ± 3.0%) compared to SHAM rats (37.7% ± 7.9%; *p* < 0.05). Additionally, the RBG% was significantly lower in OVX (23.7% ± 5.8%) compared to SHAM (34.8% ± 9.6%; *p* < 0.05) rats. Finally, the Tb.Sp% was higher in OVX (53.8% ± 7.7%) compared to SHAM (27.5% ± 14.3%; *p* < 0.05) rats. In conclusion, within the limitations of this study, inducing an osteoporotic condition in a rat model negatively influenced bone regeneration in the created bone defect and grafted with a xenograft material.

## 1. Introduction

Dento-alveolar as well as cranio-facial bone defects can be the consequence of trauma by accidents or surgical interventions [1]. Several grafting approaches can be applied to successfully treat such bone defects [2]. Bone grafting involves using natural or synthetic bone substitutes to stimulate healing of bone through three different mechanisms: osteogenesis, osteoconduction and osteoinduction [3]. Osteogenesis is the formation of new bone by osteoblastic cells present within the graft material, osteoconduction is the ability to support the growth of bone over its surface and osteoinduction is the ability to induce differentiation of pluripotential stem cells from surrounding tissue to an osteoblastic phenotype [4]. For instance, autogenic bone grafting is considered the “gold standard”, but it is associated with problems of availability and it needs a second surgical procedure [1,5]. In contrast, allograft obtained from bone bank is easily available but has a significant risk of disease transmission [2,6]. Therefore, xenograft and synthetic bone substitutes are the most commonly used graft materials in dental clinics [6]. Among them, anorganic bovine bone (e.g., Bio-Oss or similar products) is perhaps the most widely used and considered as reference material by notified bodies involved in medical device registration [7,8].

Several hard tissue alveolar ridge augmentation methods of implant therapy have been reported, including guided bone regeneration (GBR), maxillary sinus floor augmentation, vertical and/or lateral alveolar ridge augmentation and distraction. In the GBR method, barrier membrane is used [9]. A systematic review on the efficacy of barrier membranes on bone regeneration [10] showed that the use of such membranes would increase the amount of vertical augmented bone. Concerning vertical and/or lateral alveolar ridge augmentation, a Cochrane systematic review [11] showed that, although it is efficient to use a bone substitute (Bio-Oss) for horizontal bone augmentation, implants placed in bone augmented with Bio-Oss showed an increased failure rate, and the healing time was increased by three months compared with that of autogenous bone. Although the distraction technique costs more than GBR and bone grafting, it could shorten the treatment period, so this technique is effective for vertical ridge augmentation. However, the distraction technique is hard to apply to thin knife-edge bone. In any case, vertical augmentation techniques are associated with a higher complication rate [9]. Bone augmentation procedures are advanced surgical interventions. Growth factors that promote healing and regeneration are mostly used along with the grafting materials since there are multiple factors affecting the treatment outcomes. Growth factors used in dentistry are divided into platelet concentrates and recombinant growth factors. These agents are broadly used when bone-healing mechanisms are affected by the patient’s medical conditions. Guided bone regeneration and autogenous block bone grafting are two of the well-documented and safely applicable augmentation techniques [12]. The recent Food and Drug Administration approval of recombinant human bone morphogenetic proteins (rhBMPs) has given clinicians an added treatment option for reconstructing localized and large jaw defects. Currently, several patients have been successfully treated with the combination of bone graft and rhBMP-2 and the results have been documented as predictable and safe by clinical and radiologic examinations follow-up. A systematic review on the rhBMP-2 application in craniomaxillofacial reconstruction defects evaluated the real efficacy and safety of BMPs [13]. According to the results, the use of different BMPs can positively influence the surgery. In addition, there are no statistically significant differences between the use of biomaterials added with BMP and not. The study data confirm the excellent documents about the possible combination of using substitute materials and growth factor for treating large and minor craniofacial bone defects [13].

Although anorganic bovine bone products perform well in healthy patients, limited information is available about their performance under compromised conditions, e.g. diabetes and osteoporosis [14]. In patients suffering from these systemic diseases, bone healing can be very unpredictable [15]. As demonstrated in previous studies, an osteoporotic condition may delay bone healing, increase resorption of bone materials and decrease the rate of bone ingrowth [16,17]. A common observation associated with osteoporosis is that the bone formation by osteoblasts is decreased. This is in part due to the lower proliferation/differentiation rate of mesenchymal stem cells (MSCs) into mature osteoblasts [18]. Suppression of MSCs in osteoporotic bone can be the consequence of a decreased synthesis of specific osteogenic-related factors [18]. Further, increased osteoclast activity is also commonly observed in osteoporotic bone [19].

Despite available knowledge on osteoporosis, its influence on bone regeneration in relation to bone grafting is still less understood. In addition, no information is available about how osteoporosis affects bone regeneration in grafted defects if osteoporosis onset is after graft installation.

The success of alveolar ridge bone augmentation treatment is associated first with their early integration, and then for their long-term performance on the maintenance of bone graft healing. Although there are several studies involving bone grafting healing in a well-established osteoporotic condition, information about the effect of altered bone metabolism on the bone grafting healing after establishment of bone grafting healing is scarce. While it is apparent that bone augmentation in osteoporotic patients has a risk of failure for new bone regeneration, it can be hypothesized that such alteration of bone metabolism developed after bone graft placement can also negatively affect bone regeneration [20]. However, there is no scientific literature reporting on the effect of distribution of bone metabolism on bone graft healing characteristics of already well-integrated bone graft.

Clinically, the mission of regenerative dentistry is restoring damaged alveolar bone in both healthy and medically compromised patients. The key step in alveolar bone regeneration is to stimulate a cascade of healing events, which can promote bone quantity and quality. Complications regarding bone regeneration in patients with systemic impaired bone metabolism (e.g., osteoporosis) represent a rapidly increasing clinical challenge.

In view of experimental procedures requiring osteoporotic conditions, several osteoporotic animal models have been described. For example, the ovariectomized (OVX) rat is commonly used model in osteoporosis-related research [16]. OVX animals exhibit loss of trabecular bone similar to humans. Furthermore, diminished trabecular bone morphology in the femoral condyle has been confirmed in OVX rats by histological examination [21]. Therefore, this OVX rat model seems appropriate to be used in studies dealing with the healing of a bone defect in relation to an osteoporotic condition.

The limitations of pre-clinical animal models should be mentioned: animal studies will only become more valid predictors of human reactions to exposures and treatments if there is both substantial improvement in their scientific methods and more systematic review of the animal literature as it evolves. Our research group published pre-clinical systematic review and meta-analysis aimed to systematically assess bone regeneration by using anti-osteoporotic drugs in adjunction with bone grafting [22]. Preclinical systematic reviews of data from preclinical literature are important for a number of reasons [23]. First, although systematic reviews are not bias free, their purpose is to reduce it by outlining transparent aims, objectives and methodology. This approach enables us to identify all of the published literature to answer a particular research question. In turn, this may highlight gaps in our knowledge which can be fulfilled by further preclinical experimentation, or it can help us avoid unnecessary replication which is unethical and of limited benefit. Systematic reviews of animal research, if they are used to inform the design of clinical trials, particularly with respect to appropriate drug dose, timing and other crucial aspects of the drug regimen, will further improve the predictability of animal research in human clinical trials and successful translational process [23].

However, there is a lack of evidence as to whether altered bone metabolism due to osteoporosis can affect bone regeneration related to bone-defect grafting. Differently, our study model was designed to replicate the effects of late-induced osteoporosis after the bone grafting procedure was performed. Considering all this, we hypothesized that an initial healing of bone-defect grafting would be affected due to metabolic bone alteration. Consequently, the purpose of this study was to utilize this animal model to investigate the effect of an osteoporotic condition on bone regeneration after initial healing of a bone defect using a xenograft material.

## 2. Materials and Methods

### 2.1. Experimental Animal Model

The present study was approved by the Animal Ethical Committee at King Saud University, College of Dentistry, Riyadh, Saudi Arabia (Approval No. 4/67/389683). All in-vivo experiments obeyed the guidelines (national and international) for animal care and conformed to the ARRIVE guidelines. The study sample comprised a total of 16 healthy female Wistar rats (age ~12 weeks and weighing around ~250 g). The animals were housed under veterinary supervision in standardized rat cages (4–5 animals per cage), maintained in a laboratory environment with controlled temperature (22 °C–24 °C), humidity (45–55%) and 12-h light and dark cycles. All the animals had ad libitum access to a standard rat chow diet and water.

### 2.2. Sample Size Calculation

The sample number estimation was calculated based on a power analysis using the following formula: n1 = n2 = n3 = 1 + 2C(s/d)^2^. We assumed a standard deviation (s) of 12.5 and an effect size (d) of 15. C-value was fixed at 7.85 (resulting from 1-β = 0.8 and α = 0.05). According to these assumptions, 6 animals were included.

### 2.3. Experimental Surgical Procedures

**Figure 1:**
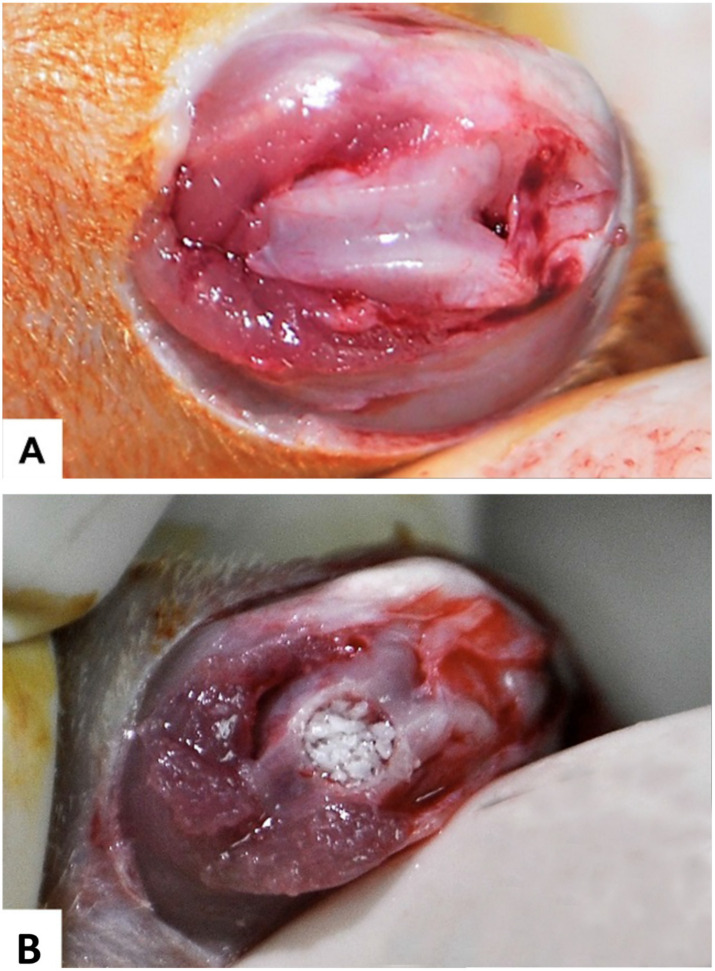
Pictures of the surgical procedure: (**A**) the femoral condyle exposed; and (**B**) bone defect filled with InterOss^®^ material.

All experimental surgical procedures were performed under general anesthesia (GA), by administering a single intraperitoneal injection comprising a combination of 0.2 mg/kg xylazine (Chanazine, Chanelle Pharmacuetical, Dublin, Ireland) and 0.5 mg/kg ketamine hydrochloride (Ketamine, Pharmazeutische Präparate, Giessen, Germany). Once the animal was anesthetized, the left leg was shaved and disinfected using Povidone-iodine 10% solution (Alphadin, MedicScience, Haryana, India). A longitudinal parapatellar skin incision, 2 cm in length, was made along the midline over the distal femoral condyle. The knee joint capsule was identified through blunt dissection of the skin flap and was incised longitudinally. The patellar ligament was elevated and retracted laterally for complete exposure of the knee joint and distal femoral condyle. Using surgical drills in a low-speed rotary drill along with saline irrigation as coolant, a cylindrical bone defect (3 mm in diameter and 3 mm in depth) paralleling the long axis of the femoral shaft was created in the intercondylar notch. Then, bone defect was filled with a xenograft material, anorganic cancellous bone graft granules, and particle size: 0.25–1 mm (InterOss^®^, SigmaGraft Inc., Fullerton, CA, USA) (Figure 1). After placement of the graft material, the soft tissue layers and skin were closed with VICRYL™ (4-0) polyglactin 910 resorbable sutures (Ethicon, Somerville, NJ, USA).

**Figure 2:**
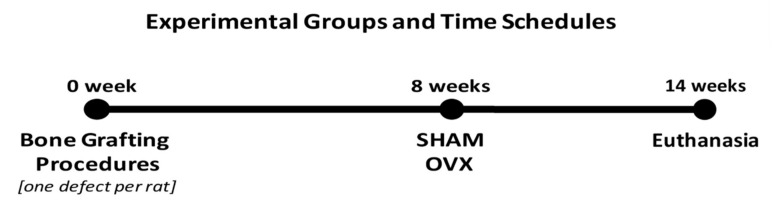
Experimental animal groups and timeline for surgical procedures and sacrifice in study animals.

After 8 weeks of bone healing, rats were randomly ovariectomized (OVX) or sham-operated (SHAM) (Table 1), as previously described [21]. Six weeks later, animals were euthanized by CO_2_-suffocation. Bone specimens were harvested and processed for histological and histomorphometric evaluation. The timelines of experimental design are described in Figure 2.

### 2.4. Histological Specimen Preparation and Evaluation

**Figure 3:**
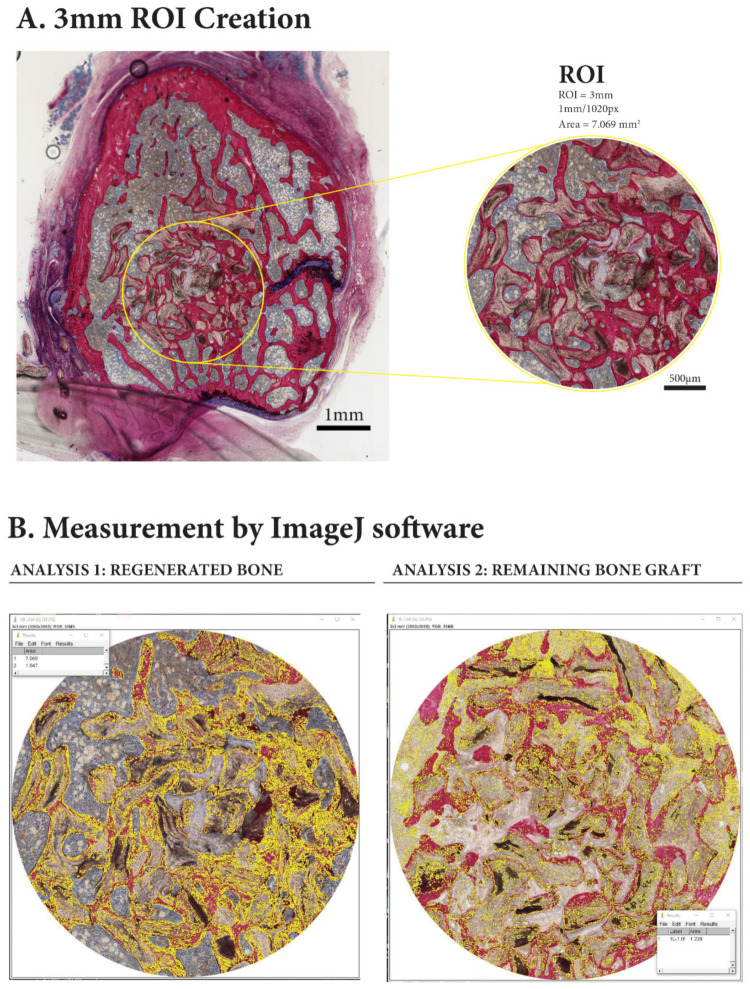
In the histological sections: (**A**) a 3-mm region of interest (ROI) was identified; and (**B**) quantitative measurements were then made to assess new bone formation (N-BF%), remaining bone graft (RBG%), and trabecular bone space (Tb.Sp%) using ImageJ software.

Harvested bone specimens were fixed in 10% neutral buffered formalin solution. The samples were dehydrated in ascending concentrations of ethyl alcohol from 70% to 100% and subsequently embedded in poly-(methyl methacrylate) (pMMA) resin, prepared by mixing 600 mL of methyl methacrylate monomer (Acros Organics BVBA, Thermo Fisher Scientific, Geel, Belgium), 60 mL dibutyl phthalate (Merck KGaA, Darmstadt, Germany) and 1.25 g perkadox (AkzoNobel, Amersfoort, Utrecht, The Netherlands). After polymerization, ~10–15 µm thick serial transverse sections (perpendicular to long axis of femur) of the resin embedded specimens were made using a diamond-coated hard-tissue microtome (Leica^®^, Microsystems SP 1600, Nussloch, Germany). The first section of each specimen was made about 1 mm below the bone surface, and then sectioning was continued distally. All sections were stained with methylene blue and basic fuchsine, as described previously [24]. Three sections, at different levels, were selected for further histological assessment. Histological and histomorphometric evaluation were carried out using a light microscope (Aperio ImageScope, Leica Biosystems, Buffalo Grove, IL, USA). The histomorphometric analyses were done using a computer-based image analysis system (IMAGE-J 1.4, National Institute of Health, Bethesda, MD, USA). Blinded histomorphometric measurements were performed for the three selected histological sections per defect (at ×10 objective magnification). First, a circular region of interest (ROI) with a 3-mm diameter equal to the created defect was identified (Figure 3). Then, within this ROI, the amount of new bone formation (N-BF%), residual graft material (RBG%) and trabecular bone space (Tb.Sp%) were determined as area percentages.

### 2.5. Statistical Analysis

All quantitative data were expressed as mean ± standard deviation (SD). Statistical analyses were performed using InStat Statistical Program (Version 3.05, GraphPad Software, San Diego, CA, USA). An unpaired Student’s *t*-test was conducted to evaluate differences in the mean values between the two study groups. The level of significance was set at 95% (*p* < 0.05).

## 3. Results

### 3.1. Animal Observations

Postoperative healing was uneventful in all animals and no complications were observed after the bone grafting and ovariectomy surgeries, except one rat (from SHAM group) that died due to GA complications. Consequently, seven rats in the SHAM group and eight rats in the OVX group were finally examined.

### 3.2. Descriptive Histological Evaluation

**Figure 4:**
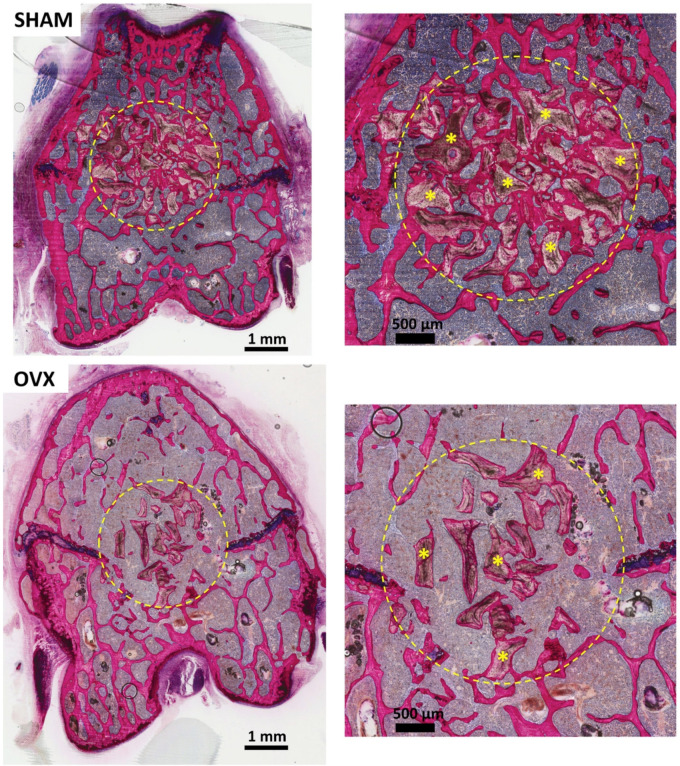
Representative histological images of pMMA sections: (**left**) for SHAM and OVX at 14 weeks post-implantation; and corresponding images at ×20 magnification (**right**). Methylene blue and basic fuchsin staining was performed. Within the ROI, images show the evident presence of new bone, remaining InterOss^®^ material (yellow*) and trabecular bone space (Tb.Sp).

Representative images of the histological sections are depicted in Figure 4. The histological sections of the OVX specimens revealed that the trabecular bone had an osteopenic appearance. The trabecular network was less dense and irregular compared to SHAM sections. At 14 weeks, images showed a higher trabecular number and less intertrabecular spacing for SHAM compared to OVX bone specimens. In between the bone trabeculae, bone marrow-like tissue was observed, characterized by the presence of mononuclear cells. As the bone tissue was stained pink, it could easily be discerned from the grafted InterOss^®^ granules. More granules seemed to remain in the defects in the SHAM animals compared to OVX animals.

**Figure 5:**
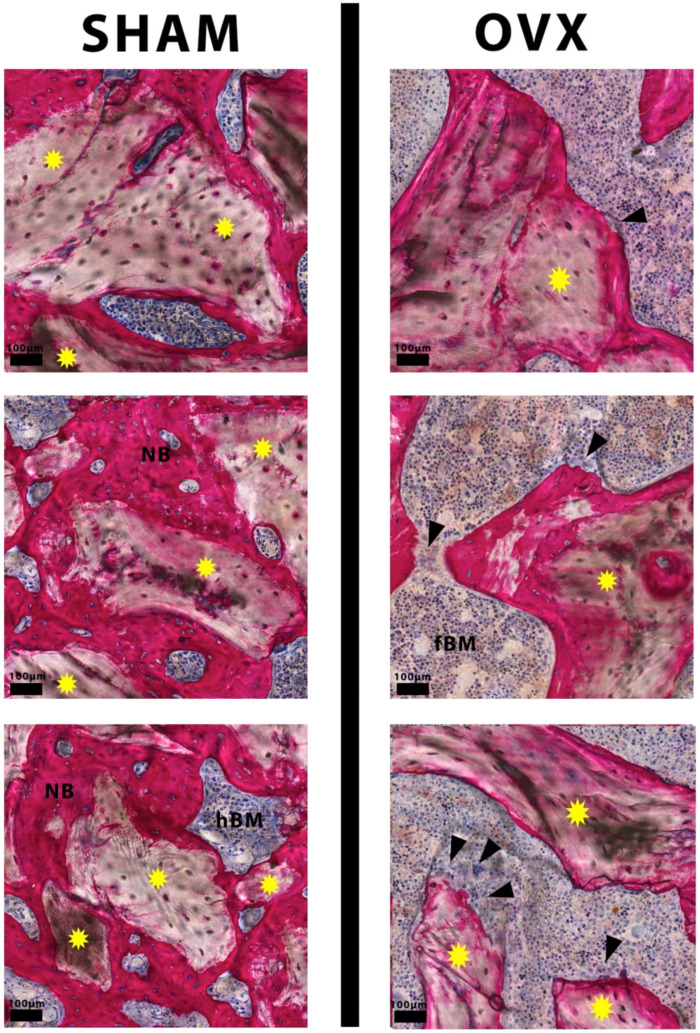
Representative histological images at higher magnification showing InterOss^®^ granules (yellow stars) well integrated and completely covered with newly formed bone (NB) in SHAM rats (**left**). In OVX rats, eroded bone surface and osteoclast-like cells (black arrowheads) were present (**right**). Bone marrow-like tissue in OVX rats contained more fat cells (fBM) compared to the hypercellular bone marrow-like tissue (hBM) in the SHAM group.

Higher magnification (Figure 5) revealed that the majority of the InterOss^®^ granules in the SHAM specimens were surrounded by abundant new bone formation, which was in direct contact with the graft material. Further, bone bridging was observed between InterOss^®^ granules. The grafted granules seemed to be completely covered by newly formed bone. High magnification images of the OVX specimen showed less newly formed bone in the osseous defects. The bone defect was for the major part filled with bone marrow-like tissue. The bone marrow-like tissue in the OVX rats contained more fat cells and less plasma cells compared to SHAM rats. Frequently, only a very superficial layer of bone was present on the surface of the granules. Occasionally, even no bone at all was seen covering the granules. Further, light micrographs showed the frequent presence of osteoclast-like cells at the interface between bone marrow-like tissue and granules (Figure 5). The images demonstrated also that some bone trabeculae in the OVX rats had an eroded appearance, which could be associated with the presence of osteoclast-like cells.

### 3.3. Histomorphometric Evaluation

**Figure 6:**
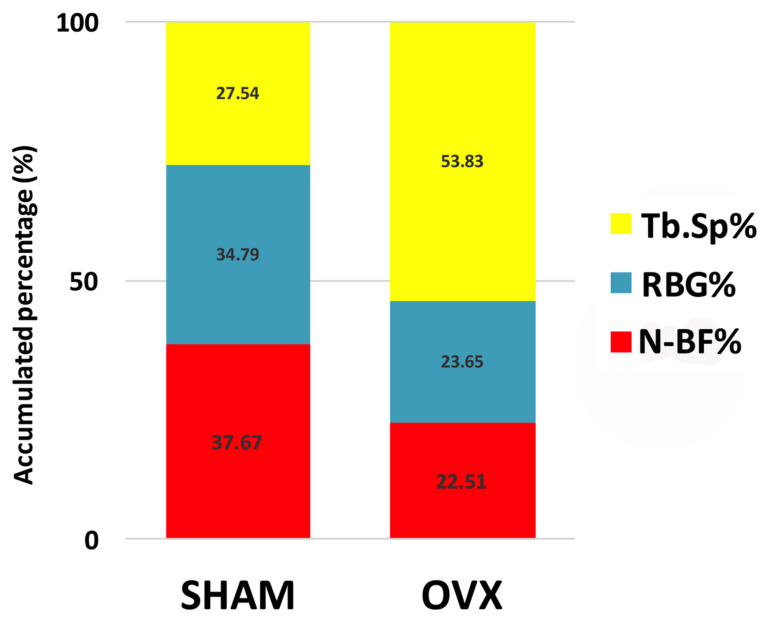
Histomorphometric evaluation of the mean volume fractions of new bone formation (N-BF%, red bar), remaining grafting material (RBG%, blue bar) and trabecular bone space (Tb.Sp%, yellow bar) occupying the defects after the 14 weeks of healing. Statistical analysis showed significant difference between SHAM and OVX for all parameters (*p* < 0.05).

The results of the histomorphometric evaluation of osseous defects grafted with InterOss^®^ granules are depicted in Table 2 and Figure 6. Data show a significantly decreased amount of new bone formation (N-BF%) in OVX rats compared to SHAM rats (*p* < 0.05). Additionally, the amount of remaining graft material (RBG%) was significantly lower in OVX compared to SHAM (*p* < 0.05). Finally, the mean of trabecular bone space (Tb.Sp%) was significantly higher in OVX compared to SHAM (*p* < 0.05).

## 4. Discussion

The present study aimed to evaluate the effect of inducing an osteoporotic condition after eight weeks of initial healing of bone defects grafted with xenograft in a rat model. The results reveal greater new bone formation within osseous defects in healthy compared to osteoporotic bone conditions. On the other hand, reduction in the remaining graft material was greater in the osteoporotic bone condition. In addition, osteoporotic bone showed larger areas of soft tissue/marrow space compared to healthy bone.

Clinically, deproteinized bovine bone graft material is extensively used to repair osseous defects [8]. Multiple human clinical studies have already been performed and long-term data regarding the outcome of bone grafting procedures have been reported. For instance, Piattelli et al. [7] conducted a histological analysis in 20 patients treated with Bio-Oss up to four years of bone healing. They concluded that anorganic bovine bone is osteoconductive and promotes the successful long-term outcome of bone grafting. In another study by Scarano et al. [25], bovine porous bone mineral for maxillary sinus augmentation was used and then dental implants installed. Histological results in an implant retrieved four years after insertion show direct contact between bone and implant without an interposition of the graft material particles. They concluded that that the slow resorption of the bovine graft particles did not jeopardize the osseointegration of the implant. Further, the results from a six-year randomized-controlled clinical trial with bovine bone material by Stavropoulos and Karring [26] show improvements in intrabony defect healing using radiographs and clinical assessment parameters.

Experiments performed with animal species such as rabbits, goats, sheep, dogs and rodents are often used to study bone regeneration. For mimicking osteoporotic bone conditions, rats were utilized in the present study to simulate alterations in bone regeneration following induced estrogen deficiency as in human. Although rats are small experimental animals and have a different bone formation and remodeling rate compared with humans, they are excellent preclinical models for studying osteoporotic changes as they closely emulate pharmaco-therapeutic response and allow studying the effect of estrogen depletion on the skeleton [27]. However, it has to be noticed that the preclinical model as used in the present study encounters several limitations that need to be solved in prospective studies. For instance, it has been noticed that ovariectomized rats have a faster bone turnover than patients with osteoporosis. Therefore, further in vivo studies can be suggested to validate the effect of osteoporosis on bone regeneration related to different bone substitutes. In addition, a larger animal model should be used to clarify the effect of osteoporosis with bone graft under challenged bone condition.

In animal models of experimental osteoporosis, the assessment of the biomaterial-mediated bone regeneration process in subcritical sized defects, considered established approaches relied on [28]. Despite the proven capability of the reported models, the usage of critical size defects, i.e., intraosseously established wounds that do not report spontaneous healing, has been considered the standard approach for the validation of translational bone regenerative strategies and output the intrinsic regenerative potential of the grafted bone. In the current study, the femoral condyle OVX rat model allowed examined bone regeneration in a 3-mm critical size defect. For instance, we recently published an in vivo experimental study using the same femoral condyle model of 3-mm critical size defect. The untreated (empty) defects did not heal without intervention, making the rat femoral condyle model a well-established and standardized critical size defect model highly useful for evaluating bone regeneration in healthy and osteoporotic bone [29].

The biological performance of deproteinized bovine bone grafts is widely investigated in preclinical studies using healthy animals [24,30,31,32,33,34]. These confirmed that bovine bone particles enhance bone regeneration, and its remnants can become integrated very well with the newly formed bone. In many histological studies, osteoblasts and osteoclasts were observed in conjunction with bovine bone particles as well as with the newly formed bone. Intimate contact between the implanted material and the newly formed bone was also commonly presented. For instance, van Houdt et al. [24] tested bovine bone (Bio-Oss) implanted in femoral condylar bone defects in rats. At 12 weeks, new bone formation in direct contact with the Bio-Oss granules was observed. The remaining Bio-Oss granules were completely covered by new bone. In line with those observations, histological analysis in the present study demonstrated similar findings in the SHAM (healthy) animals using InterOss^®^ bone granules after 14 weeks of healing (Figure 5).

Beside the grafted material, the bone condition is the key factor affecting the graft healing and its integration with new bone [16,35,36]. In many clinical cases, the presence of osteoporosis is a major challenging condition in patients undergoing bone grafting surgery due to decreased capacity of bone regeneration [16,37]. In a retrospective analysis of 49 patients, an alveolar bone grafting procedure was impaired in 11 patients due to an unfavorable bone condition (i.e., osteopenia) [37]. This is primarily due to estrogen deficiency, which negatively influences the bone metabolism [38]. Estrogen deficiency causes an imbalance in osteoblastic/osteoclastic processes and results in an increased breakdown of bone and a reduced bone formation [39].

In previous preclinical studies, ovariectomized rats were used to evaluate qualitatively and quantitatively the influence of estrogen deficiency on bone grafting [40,41]. For example, Luize et al. [41] examined bone blocks to augment a mandibular defect in OVX rats. At 7, 14 and 28 days post-surgery, histological analysis showed a delay in the osteogenic activity and bone healing in OVX rats compared to SHAM rats. The majority of grafted materials in OVX animals appeared to be interspaced by fibrous tissue. Additionally, OVX rats exhibited significantly less new bone formation compared to SHAM rats. This result is in accordance with the present study.

In contrast, some other animal studies did not observe a significant effect in terms of new bone formation related to bone grafting in osteoporotic versus healthy animals [24,42,43,44]. This discrepancy might be due to differences in the experimental design, animal model, type of biomaterials, bone defects and evaluation periods. Therefore, it is difficult to establish direct correlations between these studies.

Despite the contradiction in previous studies, the present findings sustain an impaired regenerative potential of bone grafting in OVX animals. Differently, our study model was designed in such a way to replicate the effects of late-induced osteoporosis after the bone grafting procedure was performed. At 14 weeks of bone healing, the quality and quantity of bone formation (N-BF%) was significantly decreased in the osteoporotic rats, which indicates that bone formation in a bone defect can become compromised in an osteoporotic condition, as induced post-implantation. Nevertheless, the exact involved mechanism needs to be explored further in follow-up studies.

Previously, it has been reported that a deficiency in estrogen receptor (i.e., ER-α and ER-β) expression contributes negatively to the regenerative potential in osteoporotic bone [45]. Likewise, osteoblast-related gene expression (e.g., RUNX2, BMP2, COLLAGEN I and OSTEOCALCIN) were significantly decreased in OVX animals in relation to bone-biomaterial regeneration [30,46]. Further, the osteogenic differentiation of mesenchymal stem cells (MSCs) derived from osteoporotic bone was significantly altered [18]. This emphasizes the effect of intrinsic deficiencies in the regenerative capability of osteoporotic bone, particularly in the presence of bone biomaterials. Previous data also verify an increase of the adipogenic activation in the bone of OVX animals [46]. This seems to disrupt the normal osteogenic function within the bone tissue [26]. In agreement, we noticed more fat bone marrow with an hypocellular appearance in OVX rats compared to healthy control rats.

It is important to note that in the current study the amount of remaining graft material was significantly less in the osteoporotic bone conditions compared to healthy bone conditions. We suppose that this is caused by the observed increased presence of osteoclast-like cells around the graft granules in the OVX animals. In addition, we assume that the eroded surfaces of trabecular bone indicate hyperactivity of osteoclastic cells in osteoporotic bone compared to healthy bone. Usually, the remodeling of bone graft material occurs in multiple phases [46]. Bone graft resorption is initiated by the function of osteoclastic cells. Osteoclast activation is directly linked to osteoblast function [35]. The role of osteoclasts is not limited to the resorption of bone. They also have a significant impact on the local recruitment and bone-forming activity of osteoblasts via so-called cell–cell “crosstalk”. Bone formation and resorption during remodeling have to be aligned carefully in a process that is called “coupling” [47]. Therefore, an imbalance of osteoclast/osteoblast activities in osteoporotic bone is connected with the decreased bone formation and increased rate of resorption, which limits the potential of bone-biomaterial incorporation, as observed in our study.

The performance of bone-biomaterials for bone defect augmentation showed limitation, in regard fast resorption rate, which correlated to high number osteoclast (resorbing) cells. One possible way to overcome the mentioned limitation incorporates biomaterial that showed lower number and activity of osteoclast cells. For instance, Westhauser et al. [48] showed that addition 45S5 bioactive glass to β-tricalcium phosphate might be a way to overcome the individual limitations of both materials by a combination of their respective strengths. They concluded that volume of combined materials during the 10-week implantation period remained almost unchanged correlating with significantly decreased physical presence and less pronounced genetic activity of bone-resorbing cell populations (TRAP+). Furthermore, the presence and activity of osteoclasts is highly dependent on the chemical composition of the bone substitute material and also influences osteoblast–osteoclast crosstalk and coupling [47]. The activity of osteoclastic bone resorption can be visualized by specific staining, i.e., tartrate-resistant acid phosphatase (TRAP) staining [30,49]. Decalcification of bone specimens is required for the application of TRAP staining. TRAP staining cannot be performed using pMMA embedding. However, in our study, we were limited to examining the activity of osteoclastic cells via their TRAP expression due to low number of available specimens to prepare decalcified sections. Consequently, osteoclasts could only be analyzed by histological appearance (i.e., relatively larger and multinucleated) at higher magnification (Figure 5). In addition, proper quantification of the number of osteoclasts was not feasible. This has to be considered as a limitation of our study design.

Although the OVX rat model is a well-established preclinical model for simulating osteoporotic bone conditions, differences among species and animal models size should be considered before transferring promising findings to clinical trials with human patients. Small animal models offer the easiest way to keep animals at low costs for longer time with a high reproduction rate. The offer of markers for laboratory use is the biggest in the market. On the other hand, clinical situation is far away. The results must be interpreted very carefully and often require further studies in larger animal models. Large animal models are as close to the clinical situation as a model can be. Organs, blood supply and common physiology are relatively close to humans. Disadvantages are high costs, high personal and work effort, limitation of follow-up period and availability of markers to study special histological issues [50].

In view of the above mentioned, it is very relevant to take care of the potential implication of impaired bone regeneration in osteoporotic conditions in relation to bone grafting procedures. In these situations, a solution can be the development of bone graft material that prospectively promotes the bone healing outcome.

## 5. Conclusions

Within the limitations of this study, inducing an osteoporotic condition in rats after initial healing of a bone graft material negatively influences bone regeneration in the created bone defect. Further investigations are needed to explore the clinical implications of these findings.

## 6. Impact Statement

Recently, increasing concerns have existed about the effect of bone diseases, such as osteoporosis, on bone regeneration. However, bone-grafting complications may occur in patients with compromised medical bone condition, e.g. osteoporosis, as bone healing in such patients can be challenged or impaired. This study assessed if altered bone metabolism due to osteoporosis affects bone regeneration related to bone-defect grafting using preclinical animal models. The results suggest that inducing an osteoporotic condition in rats after initial healing of a bone graft material negatively influences bone regeneration in the created bone defect.

## Figures and Tables

**Table 1:** Study groups, material and number of animals (*n*).

Study Groups	Material	Number of Animals (*n*)
SHAM	xenograft material (anorganic cancellous bone graft granules) InterOss^®^	*n* = 7
OVX	xenograft material (anorganic cancellous bone graft granules) InterOss^®^	*n* = 8

Ovariectomized (OVX)/Sham-operated (SHAM) as control.

**Table 2:** Quantitative histomorphometric data showing mean ± SD values for new bone formation (N-BF%), remaining bone graft (RBG%) and trabecular bone space (Tb.Sp%) in the two study groups.

	SHAM (*n* = 7)	OVX (*n* = 8)
New bone formation (N-BF%)	37.7 ± 7.9 *	22.5 ± 3.0
Remaining bone graft (RBG%)	34.8 ± 9.6 *	23.7 ± 5.8
Trabecular bone space (Tb.Sp%)	27.5 ± 14.3 *	53.8 ± 7.7

* indicates *p* < 0.05.

## Data Availability

Not applicable.
